# Numerical Solution of Diffusion Models in Biomedical Imaging on Multicore Processors

**DOI:** 10.1155/2011/680765

**Published:** 2011-10-10

**Authors:** Luisa D'Amore, Daniela Casaburi, Livia Marcellino, Almerico Murli

**Affiliations:** ^1^University of Naples Federico II, Complesso Universitario M.S. Angelo, Via Cintia, 80126 Naples, Italy; ^2^SPACI (Southern Partnership of Advanced Computing Infrastructures), c/o Complesso Universitario M.S. Angelo, Via Cintia, 80126 Naples, Italy; ^3^University of Naples Parthenope, Centro Direzionale, Isola C4, 80143 Naples, Italy

## Abstract

In this paper, we consider nonlinear partial differential equations (PDEs) of diffusion/advection type underlying most problems in image analysis. As case study, we address the segmentation of medical structures. We perform a comparative study of numerical algorithms arising from using the semi-implicit and the fully implicit discretization schemes. Comparison criteria take into account both the accuracy and the efficiency of the algorithms. As measure of accuracy, we consider the Hausdorff distance and the residuals of numerical solvers, while as measure of efficiency we consider convergence history, execution time, speedup, and parallel efficiency. This analysis is carried out in a multicore-based parallel computing environment.

## 1. Introduction

High-quality images are crucial to accurately diagnose a patient or determine treatment. In addition to requiring the best images possible, safety is a crucial consideration. Many imaging systems use X-rays to provide a view of what is beneath a patient's skin. X-ray radiation levels must be kept at a minimum to protect both patients and staff. As a result, raw image data can be extremely noisy. In order to provide clear images, algorithms designed to reduce noise are used to process the raw data and extract the image data while eliminating the noise. In video imaging applications, data often have to be processed at rates of 30 images per second or more. Filtering noisy input data and delivering clear, high-resolution images at these rates require tremendous computing power. This gave rise to the need of developing high-end computing algorithms for image processing and analysis which are able to exploit the high performance of advanced computing machines. 

 In this paper, we focus on the computational kernels which arise as basic building blocks of the numerical solution of medical imaging applications described in terms of partial differential equations (PDEs) of parabolic/hyperbolic type. Such PDEs arise from the scale-space approach for description of most inverse problems in imaging [1]. One of the main reasons for using PDEs to describe image processing applications is that PDE models preserve the intrinsic locality of many image processing operations. Moreover, we can rely on standard and up-to-date literature and software about basic computational issues arising in such case (such as the construction of suitable discretization schemes, the availability of a range of algorithmic options, and the reuse of software libraries that allow the effective exploitation of high-performance computing resources). Finally, PDEs appear to be effectively implemented on advanced computing environments [2].

We consider two standard discretization schemes of nonlinear time-dependent PDEs: semi-implicit scheme and fully implicit scheme [3]. The former leads to the solution of a linear system at each time (scale) step, while the computational kernel of the fully implicit scheme is the solution of a nonlinear system, to be performed at each time (scale) step. Taking into account that we aim to solve such problems on parallel computer in a scalable way, in the first case, we use, as linear solver, Krylov iterative methods (GMRES) with algebraic multigrid preconditioners (AMG) [4, 5]. Regarding the fully implicit scheme, we use the Jacobian-Free Newton-Krylov (JFNK) method as nonlinear solver [6]. 

In recent years, multicore processors are becoming dominant systems in high-performance computing [7]. We provide a multicore implementation of numerical algorithms arising from using the semi-implicit and the implicit discretization schemes of nonlinear diffusion models underlying most problems in image analysis. Our implementation is based on parallel PETSc (Portable Extensible Toolkit for Scientific Computation) computing environment [8]. Parallel software uses a distributed memory model where the details of intercore communications and data managements are hidden within the PETSc parallel objects.

The paper is organized as follows. In Section 2, an overview of the PDE model equation used in describing some of inverse problems in imaging applications will be given. Then, the segmentation problem of medical structures is discussed. Numerical approach will be introduced in Section 3.  Section 4 is devoted to the discussion of numerical algorithms based on semi-implicit and implicit numerical schemes. In Section 6, we describe the experiments that we carried out to show both the accuracy and the performance of these algorithms, while Section 7 concludes the work.

## 2. Diffusion Models Arising in Medical Imaging

The task in medical imaging is to provide in a noninvasive way information about the internal structure of the human body. The basic principle is that the patient is scanned by applying some sort of radiation and its interaction with the body is measured. This result is the data, whose origin has to be identified. Hence, we face an inverse problem. Most medical imaging problems lead to ill-posed (inverse) problems in the sense of Hadamard [9–11]. A standard approach for dealing with such intrinsic instability is to use additional information to construct families of approximate solution. This principle characterizes regularization methods that, starting from the milestone Tikhonov regularization [12], are now one of the most powerful tools for solution of inverse ill-posed problems. In 1992, Rudin et al. introduced the first nonquadratic regularization functional (i.e., the total variation regularization) [13] to denoise images. Moreover, the authors derive the Euler-Lagrange equations as a time-dependent PDE. In the same years Perona and Malik introduced the first nonlinear multiscale analysis [14]. 

 Scale-space theory has been developed by the computer vision community to handle the multiscale nature of image data. A main argument behind its construction is that if no prior information is available about what are the appropriate scales for a given data set, then the only reasonable approach for a vision system is to represent the input data at multiple scales. This means that the original image *u*(**x**), **x** ∈ *ℜ*^2^ should be embedded into a one-parameter family of derived images, in which fine-scale structures are successively suppressed:

(1) $$SS_{\tau }:\tau \in ℜ\to u\left(x,\tau \right).$$

A crucial requirement is that structures at coarse scales in the multiscale representation should constitute simplifications of corresponding structures at finer scales—they should not be accidental phenomena created by the method for suppressing fine-scale structures. A main result is that if rather general conditions are imposed on the types of computations that are to be performed, then convolution by the Gaussian kernel and its derivatives is singled out as a canonical class of smoothing transformations [15, 16]. 

 A strong relation between regularization approaches and the scale-space approach exists via the Euler-Lagrange equation of regularization functionals: it consists of a PDE of parabolic/hyperbolic (diffusion/advection) type [17], defined as follows.

**Nonlinear Diffusion Models**

Let **x** = (*x*, *y*) ∈ *ℜ*^2^ and *u*(**x**, *τ*), defined in [0, *T*] × *Ω*, be the scale-space representation of the brightness function image *u*(**x**) defined in *Ω* ⊂ *ℜ*^2^ describing the real (and unknown) object and *u*_0_(**x**) the observed image (the input data). Let us consider the following PDE problem:

(2) $$\begin{array}{c} \frac{\partial u\left(x,\tau \right)}{\partial \tau }=\left|\nabla u\right|\nabla \cdot \left(g\left(u\right)\frac{\nabla u}{\left|\nabla u\right|}\right)\;\tau \in \left[0,T\right],\;\left(x,y\right)\in \Omega \\ u\left(0,x\right)=u_{0}\left(x\right)\;\tau =0,\;\left(x,y\right)\in \Omega . \end{array}$$

[0, *T*] is the scale (time) interval; *g*(*v*) is a nonincreasing real valued function (for *v* > 0) which tends to zero as *v* → *∞*. Initial and boundary conditions will be provided according to the problem to be solved (denoising, segmentation, deblurring, registration, and so on).

Equations in (2) describe the motion of a curve (a moving front) with a speed depending on a local curvature. Such equations, known as level set equations, were first introduced in [18]. The original idea behind the level set method was a simple one. Given an interface Γ in *ℜ*^*n*^ of codimension one (i.e., its dimension is *n* − 1), bounding an (perhaps multiply connected) open region *Ω*, we wish to analyze and compute its subsequent motion under a velocity field $\vec{v}$. This velocity can depend on position, time, the geometry of the interface (e.g., its normal or its mean curvature), and the external physics. The idea, as devised in 1988 by Osher and Sethian, is merely to define a smooth (at least Lipschitz continuous) function *ϕ*(*x*, *t*), that represents the interface Γ as the set where *ϕ*(*x*, *t*) = 0. Thus, the interface is to be captured for all later time, by merely locating the set Γ(*t*) for which *ϕ* vanishes. The motion is analyzed by convecting the *ϕ* values (levels) with the velocity field *v*. This elementary equation is

(3) $$\frac{\partial \phi }{\partial t}+v\cdot \nabla \phi =0.$$

Actually, only the normal component of *v* is needed:

(4) $$v_{N}=v\cdot \frac{\nabla \phi }{\left|\nabla \phi \right|},$$

and the motion equation becomes

(5) $$\frac{\partial \phi }{\partial t}+v_{N}\cdot \left|\nabla \phi \right|=0.$$

Taking into account that the mean curvature of Γ(*t*) is

(6) $$\text{cur}=-\nabla \cdot \left(\frac{\nabla \phi }{\left|\nabla \phi \right|}\right),$$

equation (5) describes the motion of Γ(*t*) under a speed *v*_*N*_ proportional to its curvature cur (Mean Curvature Motion, MCM equation) [18–20]. This basic model has received a lot of attention because of its geometrical interpretation. Indeed, the level sets of the image solution or level surfaces in 3D images move in the normal direction with a speed proportional to their mean curvature. In image processing, equations like (5) arise in nonlinear filtration, edge detection, image enhancement, and so forth, when we are dealing with geometrical features of the image-like silhouette of object corresponding to level line of image intensity function. Finally, the level set approach instead of explicitly following the moving interface itself takes the original interface Γ and embeds it in higher dimensional scalar function *u*, defined over the entire image domain. The interface Γ is now represented implicitly as the zeroth level set (or contour) of this function, which varies with space and time (scale) using the partial differential equation in (2), containing terms that are either hyperbolic or parabolic. The theoretical study of the PDE was done by [21] which proved existence and uniqueness of viscosity solutions.

### 2.1. A Case Study: Image Segmentation

In this paper, we use equations (2) for image segmentation. The task of image segmentation is to find a collection of nonoverlapping subregions of a given image. In medical imaging, for example, one might want to segment the tumor or the white matter of a brain from a given MRI image.

 The idea behind level set (also known implicit active contours, or implicit deformable models) for image segmentation is quite simple. The user specifies an initial guess for the contour, which is then moved by image-driven forces to the boundaries of the desired objects. More precisely, the input to the model is a user-defined point-of-view *u*_0_, centered in the object we are interested in segmenting. The output is the function *u*(**x**, *τ*). Function *u*(**x**, *τ*) in (2) is the *segmentation function, u*_0_ represents the initial *contour (initial state of the segmentation function)*, and the image to segment is *I*^0^. Moreover, as proposed in [22], instead of following evolution of a particular level set of *u*, the PDE model follows the evolution of the entire surface of *u* under speed law dependent on the image gradient, without regard to any particular level set. Suitably chosen, this flow sharpens the surface around the edges and connects segmented boundaries across the missing information. In [22, 23], the authors formalized such model as the *Riemannian mean curvature flow* where the variability in the parameter *ϵ* also improves the segmentation process and provides a sort of regularization. Thus, (2) becomes

(7) $$\begin{array}{cc} \frac{\partial u\left(x,\tau \right)}{\partial \tau } & =\sqrt{\epsilon^{2}+{\left|\nabla u\right|}^{2}}\nabla \cdot \left(g\left(\left|\nabla I^{0}\right|\right)\frac{\nabla u}{\sqrt{\epsilon^{2}+{\left|\nabla u\right|}^{2}}}\right)\\ & \;\;\;\;\;\;\;\tau \in \left[0,T\right],\;\;x=\left(x,y\right)\in \Omega \\ & u\left(x,0\right)=u_{0}\left(x\right)\;\tau =0,\;\;x=\left(x,\;y\right)\in \Omega \\ & u\left(\tau ,\;x\right)=\;0\;\tau \in \left[0,T\right],\;\;x=\left(x,y\right)\in \partial \Omega \end{array}$$

accompanied with initial condition *u*_0_ and zero Dirichlet boundary conditions. Regarding *u*_0_, it is usually defined as a circle completely enclosed inside the region that one wish to segment. 

 The term *g*(*v*), called *edge detector*, is a nonincreasing real function such that *g*(*v*) → 0 while *v* → *∞*, and it is used for the enhancement of the edges. Indeed, it controls the speed of the diffusion/regularization: if ∇*u* has a small mean in a neighborhood of a point **x**, this point **x** is considered the interior point of a smooth region of the image and the diffusion is therefore strong. If ∇*u* has a large mean value on the neighborhood of **x**, **x** is considered an edge point and the diffusion spread is lowered, since *g*(*v*) is small for large *v*. A popular choice in nonlinear diffusion models is the Perona and Malik function [14]: *g*(*v*) = 1/(1 + *v*^2^/*β*), *β* > 0. In many models, the function *g*(|∇*u*|) is replaced by its smoothed version *g*(|∇*G*_*σ*_∗*u*|), where *G*_*σ*_ is a smoothing kernel, for example, the Gauss function, which is used in presmoothing of image gradients by the convolution. For shortening notations, we will use abbreviation

(8) $$g=g\left(\left|\nabla G_{\sigma }*u\right|\right).$$

In conclusion, we use the *Riemannian mean curvature flow*, as model equation of the segmentation of medical structures: given *I*_0_, the initial image and *u*_0_ equals to a circle contained inside an object of the image *I*_0_, we are interested in segmenting, we compute *u*(**x**, *τ*) by solving (7). The level sets of *u*(**x**, *τ*), at steady state, provide approximations of the contour to detect.

## 3. Numerical Schemes

Nonlinear PDE in (7) can be expressed in a compact way as

(9) $$\frac{\partial u\left(x,y,\tau \right)}{\partial \tau }=F\left[u\left(x,y,\tau ,\nabla u\left(x,y,\tau \right),I_{0}\right)\right],$$

where

(10) $$F=\sqrt{\epsilon^{2}+{\left|\nabla u\right|}^{2}}\nabla \cdot \left(g\left(\left|\nabla I^{0}\right|\right)\frac{\nabla u}{\sqrt{\epsilon^{2}+{\left|\nabla u\right|}^{2}}}\right).$$

**Scale Discretization**

That is discretization with respect to *τ*. If [0, *T*] is the scale interval and *nscales* is the number of scale steps, we denote by *τ*_*i*_ the *i*th scale-step for all *i* = 1,…, *nscales*, so that *τ*_*i*+1_ = *τ*_*i*_ + Δ*τ*, where Δ*τ* = *T*/*nscales* is the step-size.

Using the Euler forward finite difference scheme to discretize the scale derivative on the left hand side of (9), we get

(11) $$\frac{u\left(x,y,\tau_{i}\right)-u\left(x,y,\tau_{i-1}\right)}{\Delta \tau }=F\left[u\left(x,y,\tau \right)\right]$$

or, equivalently

(12) $$u\left(x,y,\tau_{i}\right)=u\left(x,y,\tau_{i-1}\right)+\Delta \tau \cdot F\left[u\left(x,y,\tau \right)\right].$$

Let us denote as *u*_*i*_ = *u*(*x*, *y*, *τ*_*i*_), *i* = 1,…, *nscales*, the function *u* evaluated at *τ*_*i*_. Equation (12) is rewritten as

(13) $$u_{i}=u_{i-1}+\Delta \tau \cdot F\left(u\left(x,y,\tau \right)\right)\equiv G\left[u\left(x,y,\tau \right)\right].$$

Depending on the collocation value, used to evaluate *u*(*x*, *y*, *τ*) with respect to the parameter *τ*, inside the *F* function on the right hand side of (12) three iterative schemes derive:

- *explicit scheme*: *u*_*i*_ = *G*[*u*_*i*−1_], that is, the function *F* is evaluated at *u*_*i*−1_ = *u*(*x*, *y*, *τ*_*i*−1_);
- *semi-implicit scheme*: *u*_*i*_ = *G*[*u*_*i*−1_, *u*_*i*_], that is, we use *u*_*i*_ to discretize the numerator |∇*u*| of the fraction $\nabla u/\sqrt{\epsilon^{2}+|\nabla u{|}^{2}}$. Other quantities are evaluated at *u*_*i*−1_;
- *implicit scheme*: *u*_*i*_ − *G*[*u*(_*i*_)] = 0, that is, the function *F* is evaluated at *u*_*i*_.

In summary, the difference between the semi-implicit and the implicit scheme relies on the scale discretization of the term |∇*u*| at the numerator of $\nabla u/\sqrt{\epsilon^{2}+|\nabla u{|}^{2}}$ inside the function *F*. This term controls the diffusion process, and it plays the role of *edge-enhancement*. If we consider the three-dimensional (3D) domain *Ω*_*T*_ = *Ω* × [0, *T*], the semi-implicit scheme employs a sort of 2D + 1 discretization of *Ω*_*T*_ proceeding along *nscales* two dimensional (2D) slices each one obtained at *τ* ≡ *τ*_*i*_, while the fully implicit scheme uses a fully 3D discretization of *Ω*_*T*_. This difference suggests that the fully implicit scheme may provide a more accurate edge detection than the semi-implicit scheme. This difference is highlighted by considering their discretization errors.

**Space Discretization**

That is discretization with respect to (*x*, *y*). If *Ω* is the space domain, we introduce a rectangular uniform grid on *Ω* consisting of *N*_*x*_ × *N*_*y*_ (for simplicity we assume that *Ω* is a rectangular of dimension 1 × 1; this means that *h*_*x*_ = 1/*N*_*x*_ and *h*_*y*_ = 1/*N*_*y*_), nodes (*x*_*i*_, *y*_*j*_) = (*l*Δ*x*, *m*Δ*y*),*l* = 1,…, *N*_*x*_, *m* = 1,…, *N*_*y*_, and we use finite volumes to discretize the partial derivatives of *u*, as in [24, 25].

**Scale-Space Discretization**

Let

(14) $$\begin{array}{c} u_{i}^{l,m}=u\left(x_{l},y_{m},\tau_{i}\right)\in {ℜ}^{N_{x}\times N_{y}\times nscales} \end{array}$$

be the vector obtained from the scale-space discretization of the function *u*, we have the following iteration formulas.

- Explicit scheme:
  (15) $$\begin{array}{cc} \frac{u_{i}^{l,m}-u_{i-1}^{l,m}}{\Delta \tau } & =\sqrt{\epsilon^{2}+{\left|\nabla u_{i-1}^{l,m}\right|}^{2}}\nabla \\ & \;\cdot \left(g\left(\left|\nabla I^{0}\right|\right)\frac{\nabla u_{i-1}^{l,m}}{\sqrt{\epsilon^{2}+{\left|\nabla u_{i-1}^{l,m}\right|}^{2}}}\right)\Leftrightarrow \\ u_{i}^{l,m} & =\left(I+\Delta \tau {\left[A\right]}_{i-1}^{l,m}\right)u_{i-1}^{l,m}\;\forall i=1,2,\ldots ,N_{E}, \end{array}$$
  where, for each *i*, the matrix [*A*]_*i*−1_^*l*,*m*^ ∈ *ℜ*^*N*_*x*_^2^×*N*_*y*_^2^^ and *I* ∈ *ℜ*^*N*_*x*_^2^×*N*_*y*_^2^^ is the unit matrix, while *N*_*E*_ is the scale steps number.

- Semi-implicit scheme:
  (16) $$\begin{array}{cc} \frac{u_{i}^{l,m}-u_{i-1}^{l,m}}{\Delta \tau } & =\sqrt{\epsilon^{2}+{\left|\nabla u_{i-1}^{l,m}\right|}^{2}}\nabla \\ & \;\cdot \left(g\left(\left|\nabla I^{0}\right|\right)\frac{\nabla u_{i}^{l,m}}{\sqrt{\epsilon^{2}+{\left|\nabla u_{i-1}^{l,m}\right|}^{2}}}\right)\Leftrightarrow \\ u_{i}^{l,m} & =u_{i-1}^{l,m}+\Delta \tau {\left[A\right]}_{i-1}^{l,m}u_{i}^{l,m}\Leftrightarrow \\ \left(I+\Delta \tau {\left[A\right]}_{i-1}^{l,m}\right)u_{i}^{l,m} & =u_{i-1}^{l,m}\;\forall i,i=1,\ldots ,N_{\text{SI}} \end{array}$$
  where, for each *i*, the matrix [*A*]_*i*−1_^*l*,*m*^ ∈ *ℜ*^*N*_*x*_^2^×*N*_*y*_^2^^ and *I* ∈ *ℜ*^*N*_*x*_^2^×*N*_*y*_^2^^ is the unit matrix and *N*_SI_ is the scale steps number.
- Fully-implicit scheme:
  (17) $$\begin{array}{cc} \frac{u_{i}^{l,m}-u_{i-1}^{l,m}}{\Delta \tau } & =\sqrt{\epsilon^{2}+{\left|\nabla u_{i}^{l,m}\right|}^{2}}\nabla \\ & \;\cdot \left(g\left(\left|\nabla I^{0}\right|\right)\frac{\nabla u_{i}^{l,m}}{\sqrt{\epsilon^{2}+{\left|\nabla u_{i}^{l,m}\right|}^{2}}}\right)\Leftrightarrow \\ u_{i}^{l,m} & =u_{i-1}^{l,m}+\Delta \tau {\left[A\right]}_{i}^{l,m}\left(u_{i}^{l,m}\right)\;\forall i=1,\ldots ,N_{I}, \end{array}$$
  where *N*_*I*_ is the scale steps number and [*A*]_*i*_^*l*,*m*^, for each *i*, is a nonlinear vector operator on *ℜ*^*N*_*x*_^2^×*N*_*y*_^2^^, depending on *u*_*i*_^*l*,*m*^.

In particular, we apply the Crank-Nicholson scheme [3] which uses the average of the forward Euler method at step *i* − 1 and the backward Euler method at step *i*:

(18) $$\begin{array}{cc} \frac{u_{i}^{l,m}-u_{i-1}^{l,m}}{\Delta \tau } & =\frac{1}{2}[\sqrt{\epsilon^{2}+{\left|\nabla u_{i}^{l,m}\right|}^{2}}\nabla \\ & \;\;\;\cdot \left(g\left(\left|\nabla I^{0}\right|\right)\frac{\nabla u_{i}^{l,m}}{\sqrt{\epsilon^{2}+{\left|\nabla u_{i}^{l,m}\right|}^{2}}}\right)]+\frac{1}{2}\cdots \\ & \;\cdots \cdot [\sqrt{\epsilon^{2}+{\left|\nabla u_{i-1}^{l,m}\right|}^{2}}\nabla \\ & \;\;\;\cdot \left(g\left(\left|\nabla I^{0}\right|\right)\frac{\nabla u_{i-1}^{l,m}}{\sqrt{\epsilon^{2}+{\left|\nabla u_{i-1}^{l,m}\right|}^{2}}}\right)],\\ & \;\;\;\;\;\;\;\;\;\;\;\forall i=1,\ldots ,N_{I}\\ & \Leftrightarrow u_{i}^{l,m}=u_{i-1}^{l,m}+\Delta \tau \left[B_{i-1}^{l,m}u_{i-1}^{l,m}+{\left[A\right]}_{i}^{l,m}\left(u_{i}^{l,m}\right)\right],\\ & \;\;\;\;\;\;\;\;\;\;\;\;\;\forall i=1,\ldots ,{\text{N}}_{I}, \end{array}$$

where *B*_*i*−1_^*l*,*m*^ is a matrix on *ℜ*^*N*_*x*_^2^×*N*_*y*_^2^^, depending on *u*_*i*−1_^*l*,*m*^, and [*A*]_*i*_^*l*,*m*^ is a nonlinear vector operator on *ℜ*^*N*_*x*_^2^×*N*_*y*_^2^^, depending on *u*_*i*_^*l*,*m*^.

## 4. Algorithms and Their Computational Complexity

The effectiveness of these schemes depends on a suitable balance between accuracy (scale-space discretization error), number of flop/s per iteration (algorithm complexity), and the total execution time needed to reach a prescribed accuracy (software performance).

 Let us denote the discretization error *E*_*d*_. It is

(19) $$E_{d}=O\left(h_{x}^{p}\right)+O\left(h_{y}^{p}\right)+O\left(\Delta \tau^{q}\right).$$

Explicit scheme is accurate at the first order both with respect to scale and space, that is, *p* = *q* = 1; anyway, it is the one straightforwardly computable. The computational kernel is a matrix-vector product, at every scale step. This scheme requires very small time steps in order to be stable (CFL (Courant-Friedrich-Levy) condition that guarantees the stability of the evolution), and its use is limited rather by its stability than accuracy. This constraint is practically very restrictive, since it typically leads to the need for a huge amount of iterations [3]. Semi-implicit scheme is absolutely stable for all scale steps. The accuracy, in terms of discretization error with respect to both scale and space, is of the first order, because *p* = 1, *q* = 2 [24, 25]. Crank-Nicholson provides a discretization error of second order, that is, *p* = *q* = 2, but it requires extra computations, leading to a nonlinear system of equations, at every time step, while stability is ensured for all scale steps [3]. In the following, we collect these results:

(20) $$\begin{array}{c} \text{explicit}\;\text{scheme:}\;p=q=1,\\ \text{semi-implicit}\;\text{scheme:}\;p=1,\;q=2,\\ \text{implicit}\;\text{scheme:}\;p=q=2. \end{array}$$

Then, the fully implicit scheme provides an order of accuracy greater than that provided by the others. This difference may be important in those applications of image analysis where the edges are fundamental to recognize some pathologies.

 Algorithm complexity of these schemes depends on the choice of the numerical solver. Concerning the semi-implicit scheme, we employ Krylov subspace methods, which are the most effective approaches for solving large linear systems [5]. In particular, we use Generalized Minimal RESidual method (GMRES) equipped with Algebraic multigrid (AMG) preconditioner. Such techniques are convenient because they require as input only the system matrix corresponding to the finest grid. In addition, they are suitable to implement in a parallel computing environment. For the fully implicit scheme we use the Jacobian Free Newton Krylov Method (JFNK) [6]. JFNK methods are synergistic combinations of Newton-type methods for superlinearly convergent solution of nonlinear equations and Krylov subspace methods for solving the Newton correction equations. The link between the two methods is the Jacobian-vector product, which may be probed approximately without forming and storing the elements of the true Jacobian. 

 Let us briefly describe the numerical algorithms that we are going to implement, which are based on the semi-implicit and the implicit discretization schemes, together with their complexity.

**Algorithm SI (Semi-Implicit Scheme)**

For all *i* = 1,…, *N*_SI_ solution of

(21) $$\left(I+\Delta \tau {\left[A\right]}_{i-1}^{l,m}\right)u_{i}^{l,m}=u_{i-1}^{l,m}\Leftrightarrow HS_{i-1}^{l,m}u_{i}^{l,m}=u_{i-1}^{l,m},$$

with respect to *u*_*i*_^*l*,*m*^. *HS*_*i*−1_^*l*,*m*^, for each *i*, is a matrix ∈*ℜ*^*N*_*x*_^2^×*N*_*y*_^2^^. As space derivative we use the 2nd order finite covolume discretization scheme (see [24, 25] for convergence, consistence and stability). By this way, matrix [*A*]_*i*−1_^*l*,*m*^ is a block pentadiagonal matrix with tridiagonal blocks along the main diagonal and diagonal blocks along the upper and lower diagonals.

**Algorithm I (Implicit Scheme)**

For all *i* = 1,…, *N*_*I*_ solution of

(22) $$\begin{array}{cc} u_{i}^{l,m} & =u_{i-1}^{l,m}+\Delta \tau \left[B_{i-1}^{l,m}u_{i-1}^{l,m}+{\left[A\right]}_{i}^{l,m}\left(u_{i}^{l,m}\right)\right]\\ & \Leftrightarrow HI_{i}^{l,m}\left(u_{i}^{l,m},u_{i-1}^{l,m}\right)=0, \end{array}$$

with respect to *u*_*i*_^*l*,*m*^. *HI*_*i*_^*l*,*m*^, for each *i*, is a nonlinear vector operator on *ℜ*^*N*_*x*_^2^×*N*_*y*_^2^^.

**Algorithm SI**

For each scale step, to solve the linear system (21), we employ GMRES iterative method (see Algorithm 1). Computational kernel of GMRES is a matrix-vector product. Taking into account the structure of the coefficient matrix (we assume that *N*_*x*_ = *N*_*y*_ = *N*, then *h* = 1/*N* = *h*_*x*_ = *h*_*y*_), the computational cost of GMRES is

(23) $$T_{\text{GMRES}}\left(N^{2}\right)=O\left(k_{\text{GMRES}}^{\text{SI}}\cdot 5N^{2}\right),$$

where *k*_GMRES_^SI^ is the maximum iterations of GMRES (over the scale steps). Computational complexity of Algorithm SI is

(24) $$T_{\text{SI-GMRES}}\left(N^{2}\right)=O\left(N_{\text{SI}}\cdot k_{\text{GMRES}}^{\text{SI}}\cdot 5N^{2}\right).$$

Algebraic multigrid (AMG) method follows the main idea of (geometric) multigrid (MG), where a sequence of grids is constructed from the underlying geometry with corresponding transfer operators between the grids [26]. The main idea of MG is to remove the smooth error, that cannot be eliminated by relaxation on the fine grid, by coarse-grid correction. The solution process then as usual consists of presmoothing, transfer of residuals from fine to coarse grids, interpolation of corrections from coarse to fine levels, and optional postsmoothing. In contrast to geometric multigrid, the idea of AMG is to define an artificial sequence of systems of equations decreasing in size. We call these equations *coarse-grid* equations. The interpolation operator *P*_*lv*_^*l*,*m*^ and the restriction operator *R*_*lv*_^*l*,*m*^ define the transfer from finer to coarser grids and vice versa. Finally, the operator on the coarser grid at level *lv* + 1 is defined by

(25) $$A_{lv+1}^{l,m}=R_{lv}^{l,m}A_{lv}^{l,m}P_{lv}^{l,m}.$$

The AMG method consists of two main parts, the setup phase and the solution phase. During the setup phase, the coarse-grids and the corresponding operators are defined. The solution phase consists of a multilevel iteration. The number of recursive calls, which is the number of levels *lv*, depends on the size and structure of the matrix. For our case, we use the V-cycle pattern with the FALGOUT-CLJP coarse grid selection [27]. Looking at the Algorithm SI in Table 1, the preconditioner *P* is just *A*_*lv*+1_. 

 Computational cost of each iteration of GMRES is that of the AMG preconditioner plus the matrix-vector products:

(26) $$T_{\text{AMG}+\text{GMRES}}\left(N^{2}\right)=O\left(k_{\text{GMRES}}^{\text{SI}}\left(\underset{lv\cdot N^{2}}{\underset{︸}{T_{\text{AMG}}\left(lv\right)}}+5N^{2}\right)\right),$$

then, we get:

(27) $$T_{\text{SI}+\text{AMG}+\text{GMRES}}\left(N^{2}\right)=O\left(N_{\text{SI}}k_{\text{GMRES}}^{\text{SI}}lvN^{2}\right).$$

Following picture shows a schematic description of Algorithm SI that emphasizes its main steps and the most time consuming operation, that is, the matrix vector products needed at each step of GMRES.

**Algorithm I**

For each scale step, to solve the nonlinear equations (22), we employ the Jacobian-Free Newton-Krylov (JFNK) method. JFNK is a nested iteration method consisting of at least two and usually four levels. The primary levels, which give the method its name, are the loop over the Newton method:

(28) $$HI_{i}^{l,m}\left(u_{n+1}^{l,m}\right)=0\Leftrightarrow HI_{\text{i}}^{l,m}\left(u_{n}^{l,m}\right)+J_{i}\left(u_{n}^{l,m}\right)\left(u_{n+1}^{l,m}-u_{n}^{l,m}\right)=0,$$

and the loop building up the Krylov subspace out of which each Newton step is drawn:

(29) $$J_{i}\left(u_{n}^{l,m}\right)\delta u_{n}^{l,m}=-HI_{i}^{l,m}\left(u_{n}^{l,m}\right),\;\;u_{n+1}^{l,m}=u_{n}^{l,m}+\delta u_{n}^{l,m},$$

Outside of the Newton loop, a globalization method is often required. We use line search.

Forming each element of *J* which is a matrix of dimension *N*^2^ × *N*^2^ requires taking derivatives of the system of equations with respect to *u*. This can be time consuming. Using the first order Taylor series expansion of *HI*_*i*_^*l*,*m*^(*u*_*n*_^*l*,*m*^ + *ρv*), it follows that

(30) $$\begin{array}{cc} J_{i}\left(u_{n}^{l,m}\right)\delta u_{n}^{l,m} & =\frac{\left[HI_{i}^{l,m}\left(u_{n}^{l,m}+\rho \delta u_{n}^{l,m}\right)-HI_{i}^{l,m}\left(u_{n}^{l,m}\right)\right]}{\rho }\\ & \;+O\left(\rho^{2}\right), \end{array}$$

where *ρ* is a perturbation. JFNK does not require the formation of the Jacobian matrix; it instead forms a result vector that approximates this matrix multiplied by a vector. This *Jacobian-free* approach has the advantage to provide the quadratic convergence of Newton method without the costs of computing and storing the Jacobian. Conditions are provided on the size of *ρ* that guarantee local convergence.

Algorithm 3 shows a schematic description of Algoritm I that emphasizes its main steps and the most time consuming operation, that is, evaluations of the nonlinear operator *HI*_*i*_^*l*,*m*^ at each innermost step. 

 Algorithm complexity of JFNK is

(31) $$T_{\text{JFNK}}\left(N^{2}\right)=O\left(N_{\text{New}}k_{\text{GMRES}}^{I}\left[f+O\left(N^{2}\right)\right]\right),$$

where *N*_New_ is the maximum number of Newton's steps, over the scale steps, *k*_GMRES_^*I*^ is the maximum number of GMRES iterations (computed over Newton's steps and scale steps)), *f* is the number of evaluations of *HI*_*i*_^*l*,*m*^. Finally, we get

(32) $$T_{I+\text{JFNK}}\left(N^{2}\right)=O\left(N_{I}N_{\text{New}}k_{\text{GMRES}}^{I}\left[f+O\left(N^{2}\right)\right]\right).$$

A straightforward comparison between the algorithm complexity of these algorithms shows that Algorithm SI asymptotically seems to be comparable with respect to Algorithm SI. Of course, the performance analysis must also take into account the efficiency of these two schemes in a given computing environment. Next section describes the PETSc-based implementation of these algorithms that we have developed in a multicore computing environment.

## 5. The Multicore-Based Implementation

The software has been developed using the high-level software library PETSc (Portable Extensible Toolkit for Scientific Computations) (release 3.1, March 2010) [8]. PETSc provides a suite of data structures and routines as building blocks for the implementation of large-scale codes to be used in scientific applications modeled by partial differential equation. PETSc is flexible: its modules, that can be used in application codes written in Fortran, C, and C++, are developed by means of object-oriented programming techniques.

The library has a hierarchical structure: it relies on standard basic computational (BLAS, LAPACK) and communication (MPI) kernels and provides mechanism needed to write parallel application codes. PETSc transparently handles the moving of data between processes without requiring the user to call any data transfer function. This includes handling parallel data layouts, communicating ghost points, gather, scatter and broadcast operations. Such operations are optimized to minimize synchronization overheads.

 Our parallelization strategy is based on domain decomposition: in particular, we adopt the *row-block data distribution*, which is the standard PETSc data distribution. Row-block data distribution means that blocks of dimensions *N*^2^/*p* × *N* of contiguous rows of matrices of dimension *N*^2^ × *N*^2^ are distributed among contiguous processes. By the same way, vectors of size *N* are distributed among *p* processors as blocks of size *N*/*P*. Such partitioning has been chosen because overheads, due to redistribution before the solution of the linear systems, are avoided. Further, row-block data distribution introduces a coarse grain parallelism which is best oriented to exploit concurrency of multicore multiprocessors because it does not require a strong cooperation among computing elements: each computing element has to locally manage the blocks that are assigned to it. 

 The computing platform that we consider is made of 16 blades (1 blade consisting of 2 quad core Intel Xeon E5410@2.33 GHz) Dell PowerEdge M600, equipped with IEEE double precision arithmetic. Because high performance technologies can be employed in medical applications only to the extent that the overall cost of the infrastructure is affordable, and because we consider single images of medium size, we show results obtained by using 1 blade, that is, we run the parallel algorithms on up to *p* = 8 cores of a single blade. Of course, in case of multiple images or sequences of images, the use of a greater number of cores may be interesting.

## 6. Experiments

In this section we present and discuss computational results obtained by implementing Algorithm I and Algorithm SI in a multicore parallel computing machine. Before illustrating experimental results, let us briefly describe the choice of

1. test images,
2. comparison criteria,
3. parameters selection.

**Figure 1:**
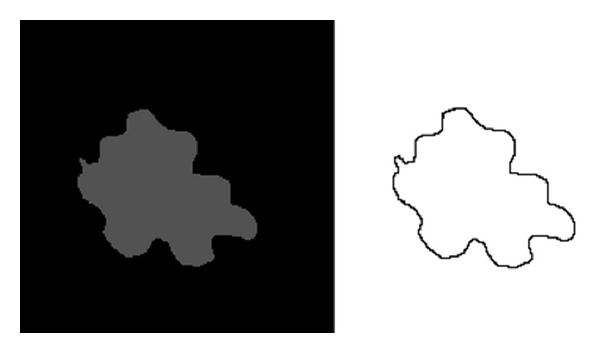
Test 1. Image size: 840 × 840, simulated image and its contour to compute.

**(1) Test Images**

we have carried out many experiments in order to analyze the performance of these algorithms. Here we show results concerning the segmentation of a (malignant) melanoma (see Figure 7) [28]. Epiluminescence microscopy (ELM) has proven to be an important tool in the early recognition of malignant melanoma [29, 30]. In ELM, halogen light is projected onto the object, thus rendering the surface translucent and making subsurface structures visible. As an initial step, the mask of the skin lesion is determined by a segmentation algorithm. Then, a set of features containing shape and radiometric features as well as local and global parameters is calculated to describe the malignancy of the lesion. In order to better validate computed results and to analyze the software performance, we first consider a synthetic test image simulating the object we are interested in segmenting (see Figure 1).

**(2) Comparison Criteria**

We compare the algorithms using the following criteria:

- distance from original solution. As measure of the difference between two curves, we use the Hausdorff distance measured between the computed curve and the original one. It is well known that the Hausdorff distance is a metric over the set of all closed bounded sets (see [31]), here we restrict ourselves to finite point sets because that is all that is necessary for segmentation algorithms [32]. Given two finite point sets *C*_1_ and *C*_2_, the Hausdorff distance *d*_*H*_ between the sets *C*_1_ and *C*_2_ is defined as follows:
  (33) $$\begin{array}{c} d_{H}\left(C_{1},C_{2}\right)=\max\left\{h\left(C_{1},C_{2}\right),h\left(C_{2},C_{1}\right)\right\},\\ h\left(C_{1},C_{2}\right)=\underset{a\in C_{1}}{\max}\;\underset{b\in C_{2}}{\min}||a-b||, \end{array}$$
  where ||·|| is the euclidean norm. It identifies the point *a* ∈ *C*_1_ that is fastest from any point of *C*_2_ and vice versa, then it keeps the maximum,
- efficiency: execution time of (serial) algorithms,
- convergence history: behavior of residuals and iteration numbers of inner solvers,
- Parallel performance: execution time, speedup, and efficiency versus cores number.

**(3) Parameters Selection**

We set *K* = 1.0 and *ϵ* = 1.0. Let us explain how we select the values of the scale step size and the number of scale steps. Regarding Δ*τ*, its value is chosen according to that required to *E*_*d*_. Taking into account that, in Algorithm SI, *E*_*d*_ is accurate at the first order with respect to Δ*τ*_SI_, while it is accurate at the second order with respect to Δ*τ*_*I*_, in Algorithm I, by requiring that the discretization error is about the same, we get

(34) $$E_{d}=O\left(\Delta \tau_{\text{SI}}\right)=O\left(\Delta \tau_{I}^{2}\right)\Rightarrow \Delta \tau_{\text{SI}}=\sqrt{\Delta \tau_{I}}.$$

Finally, in Algorithm SI, the stopping criterion of the linear solver (GMRES) uses the tolerance

(35) $$\text{TOL}=10^{-10},$$

while the preconditioner AMG uses the tolerance TOL = 10^−7^ and 25 as maximum number of AMG-levels. In the Algorithm 2, the stopping criterion of the nonlinear solver uses the tolerance

(36) $$\text{TOL}=10^{-10}.$$

Regarding the number of scale steps (*N*_SI_ and *N*_*I*_), taking into account that

(37) $$N_{\text{SI},I}=\frac{T}{\Delta \tau_{\text{SI},I}},$$

its choice depends on Δ*τ*_SI,*I*_ and on the value of *τ* ≡ *T*, that is, the value of the scale parameter corresponding to steady state of the segmentation function *u*(**x**, *τ*), solution of the PDE model. To check the steady state, we require that the residuals, corresponding to different scale steps, reach the tolerance

(38) $$\text{TOL}=10^{-9}.$$

**Figure 2:**
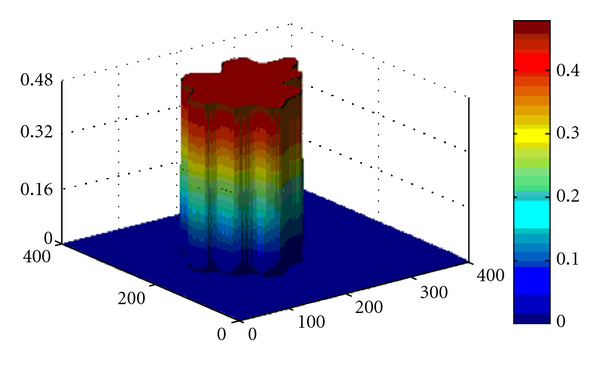
Test 1: The 3D visualization of the segmentation function *u*(*τ*, **x**) at steady state *T* = 0.4.

We found that this corresponds to *T* = 0.4 (see Figure 2) for Test 1 and to *T* = 2 for Test 2.

**Test 1: Synthetic Image**

In Tables 1, 2, and 3, we show the Hausdorff distance and execution time by requiring that discretization error is of the first, second, and third order, that is, *E*_*d*_ = *O*(10^−1^), *E*_*d*_ = *O*(10^−2^), and *E*_*d*_ = *O*(10^−3^), respectively. Hence, we get the following values of the scale step size:

(39) $$\begin{array}{c} \Delta \tau_{I}=0.4\Rightarrow \Delta \tau_{\text{SI}}=0.16,\\ \Delta \tau_{I}=0.04\Rightarrow \Delta \tau_{\text{SI}}=0.016,\\ \Delta \tau_{I}=0.004\Rightarrow \Delta \tau_{\text{SI}}=0.0016. \end{array}$$

Moreover, concerning the number of scale steps, it follows that

(40) $$\begin{array}{c} E_{d}=O\left(10^{-1}\right)\Rightarrow N_{I}=\frac{0.4}{0.4}=1,\;N_{\text{SI}}=\mathrm{int}\left[\frac{0.4}{0.16}\right]=3,\\ E_{d}=O\left(10^{-2}\right)\Rightarrow N_{\text{SI}}=\frac{0.4}{0.04}=10,\;N_{\text{SI}}=\frac{0.4}{0.016}=25,\\ E_{d}=O\left(10^{-3}\right)\Rightarrow N_{\text{SI}}=\frac{0.4}{0.04}=100,\;N_{\text{SI}}=\frac{0.4}{0.016}=250. \end{array}$$

**Figure 3:**
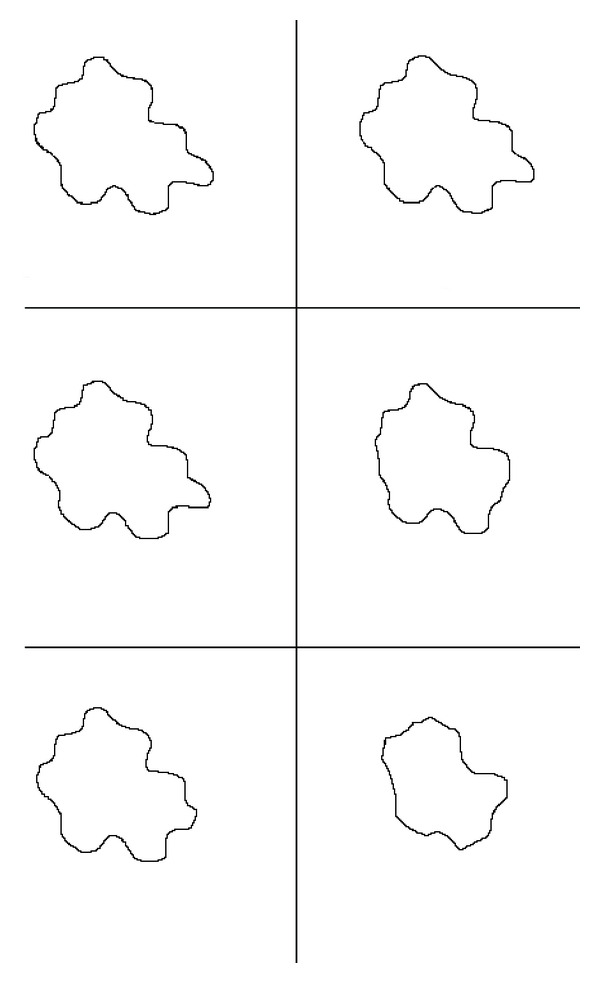
Test 1: Comparisons between segmentation results. On the left Algorithm I. On the right Algorithm SI. First row: Δ*τ*_*I*_ = 0.4, Δ*τ*_SI_ = 0.16. Second row: Δ*τ*_*I*_ = 0.04, Δ*τ*_SI_ = 0.16. Third row: Δ*τ*_*I*_ = 0.004,  Δ*τ*_*I*_ = 0.0016.

Note that while in the first case, that is, if we require *E*_*d*_ = *O*(10^−1^), these two algorithms are quite numerically equivalent, both in terms of execution time and of the computed result; as discretization error decreases, Algorithm I appears to be more robust than Algorithm SI, in the sense that Algorithm I reaches the steady state with high accuracy (the Hausdorff distance is of 95%), while the computed results of Algorithm SI are less accurate, even though the execution time of Algorithm I sometimes slightly increases. These results suggest that if it needs to get an accurate and reliable result, Algorithm I should be preferable.  Figure 3 show segmentation results. Finally, note that the execution time of these two algorithms asymptotically is the same, as stated by the analysis of computational cost carried on in Section 4.

**Convergence History**

**Figure 4:**
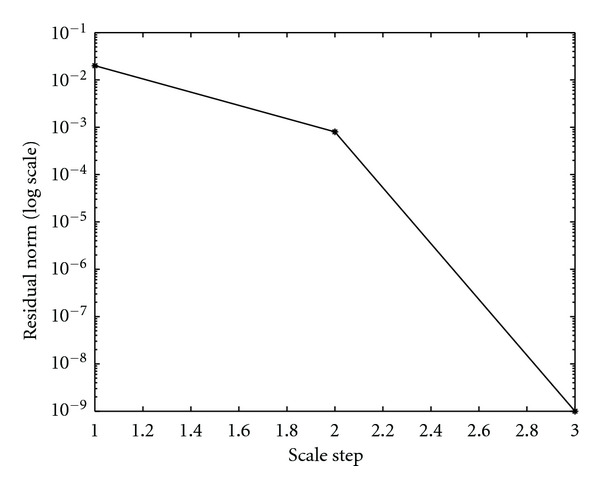
Algorithm SI. Behavior of the relative residual ||*r*_*i*_||_2_/||*r*_0_||_2_ versus 3 scale steps. Δ*τ*_SI_ = 0.16.

**Figure 5:**
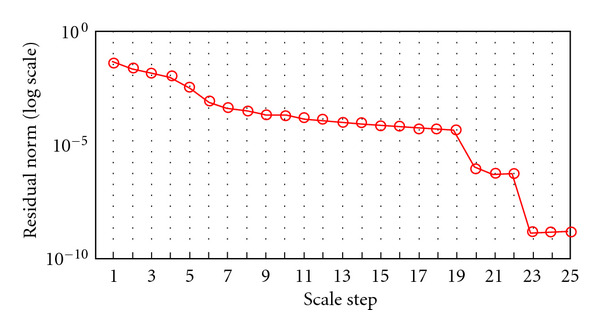
Algorithm SI. Behavior of the relative residual ||*r*_*i*_||_2_/||*r*_0_||_2_ versus 25 scale steps. Δ*τ*_SI_ = 0.016.

**Figure 6:**
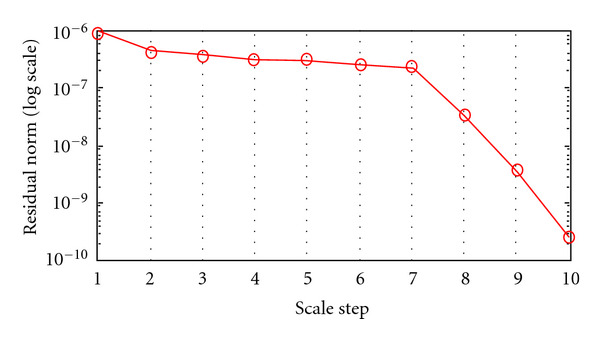
Algorithm I. Behavior of the relative residual ||*r*_*i*_||_2_/||*r*_0_||_2_ versus 10 scale steps. Δ*τ*_*I*_ = 0.04.

Convergence history is illustrated by showing the behavior of relative residuals versus the scale steps (see Figures 4, 5, and 6), and by reporting iteration number of the GMRES and of Newton's method, respectively, (see Tables 4, 5, 6, and 7). We consider Δ*τ*_SI_ = 0.16, 0.016, and Δ*τ*_*I*_ = 0.04.

**Figure 7:**
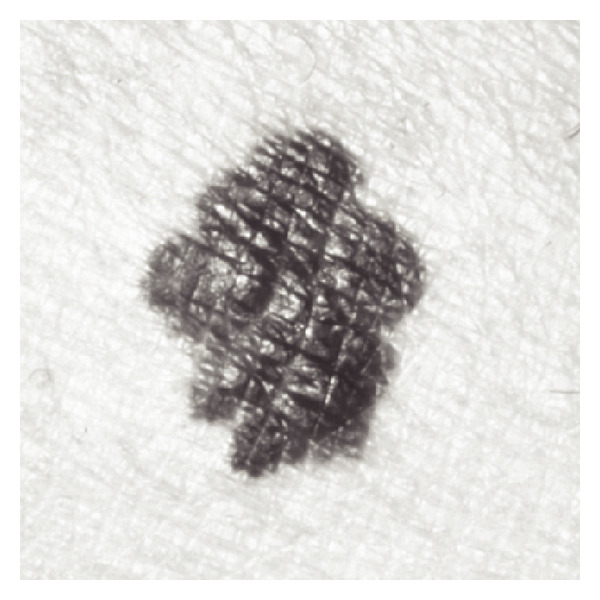
Test 2: ELM of a melanoma. Image size is 840 × 840.

**Figure 8:**
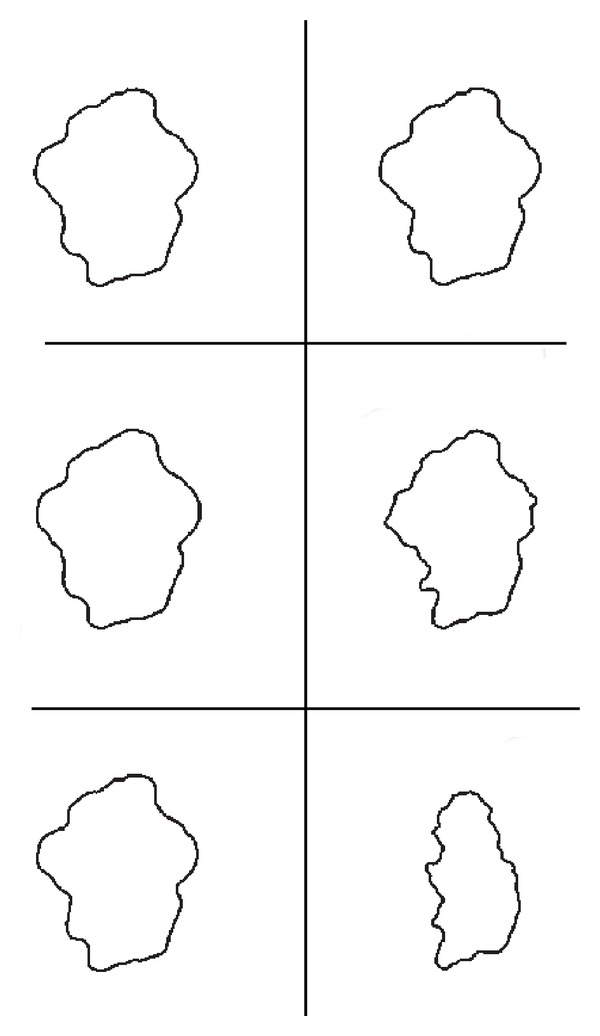
Test 2: Comparisons between segmentation results. On the left Algorithm I. On the right Algorithm SI. First row:, Δ*τ*_*I*_ = 0.4, Δ*τ*_SI_ = 0.16. Second row: Δ*τ*_*I*_ = 0.04, Δ*τ*_SI_ = 0.16. Third row: Δ*τ*_*I*_ = 0.004,Δ*τ*_*I*_ = 0.0016.

In the following, we show results corresponding to the segmentation of a melanoma (see Figure 7). As expected, because this is a real image, the steady state is reached at a scale greater than that of the synthetic test image, that is, *T* = 2, thus both algorithms require a greater number of scale steps to the reach the steady state. Tables 8, 9, and 10, and Figure 8 compare results of Algorithm SI and Algorithm I.

**Parallel Performance**

We show the performance of the multicore-based parallel algorithms and their scalability as the number of cores increases. We run the parallel algorithms using up to *p* = 8 cores of the parallel machine.

**Figure 9:**
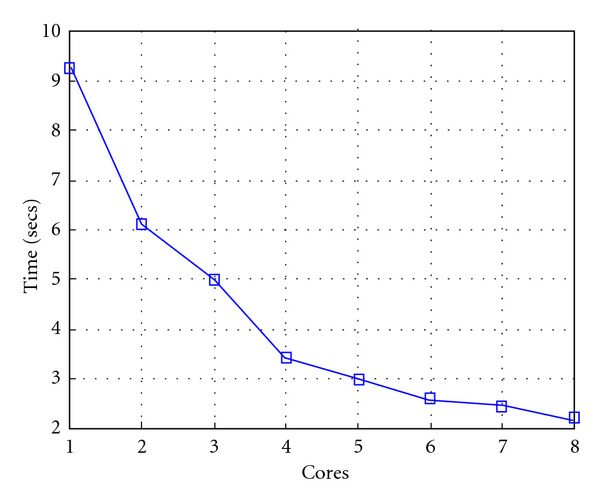
Test 1: Algorithm SI: Total execution time versus the number of cores. Δ*τ* = 0.16. 3 scale steps.

**Figure 10:**
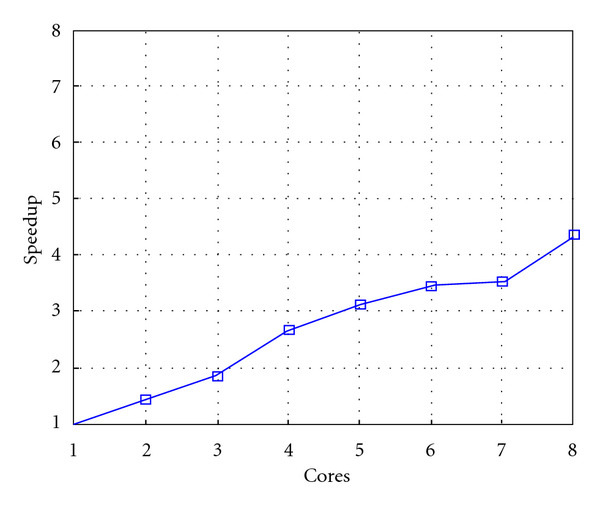
Test 1: Algorithm SI: Speedup versus the number of cores. Δ*τ* = 0.16. 3 scale steps.

**Figure 11:**
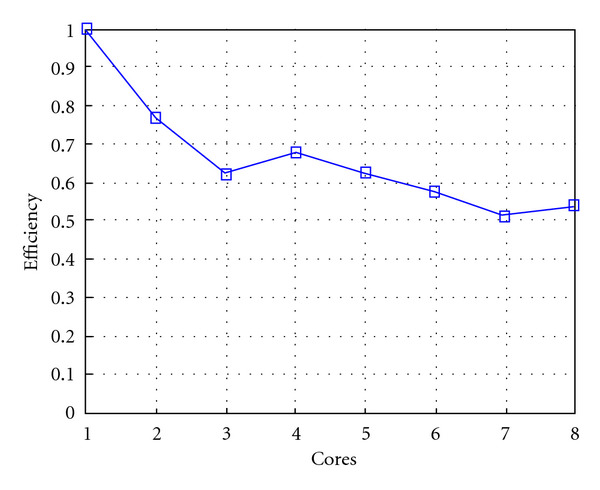
Test 1: Algorithm SI: Parallel efficiency versus the number of cores. Δ*τ* = 0.16. 3 scale steps.

**Figure 12:**
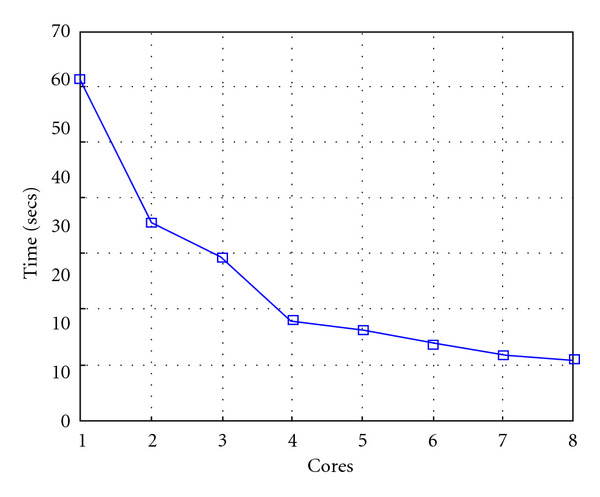
Test 1: Algorithm SI: Total execution time versus the number of cores. Δ*τ* = 0.016.

**Figure 13:**
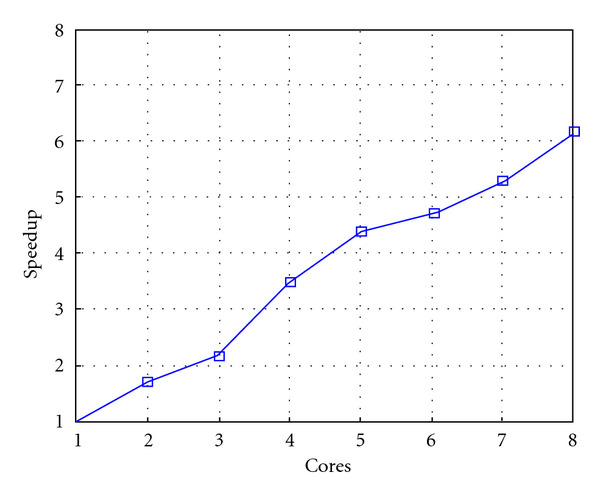
Test 1: Algorithm SI: Speedup versus the number of cores. Δ*τ* = 0.016.

**Figure 14:**
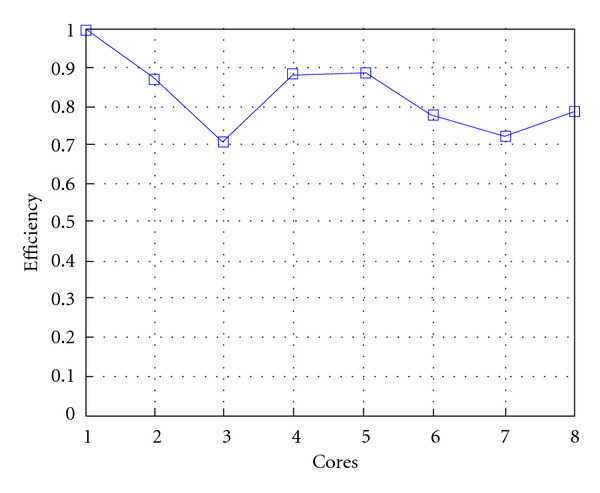
Test 1: Algorithm SI: Parallel efficiency versus the number of cores. Δ*τ* = 0.016.

Following Figures report execution time, speedup, and efficiency of Algorithm SI, at scale step size Δ*τ* = 0.16 (i.e., *E*_*d*_ = (10^−1^) (i.e., Figures 9, 10 and 11), then same results are shown at scale step size Δ*τ* = 0.016, corresponding to *E*_*d*_ = *O*(10^−2^) (i.e., Figures 12, 13 and 14).

**Figure 15:**
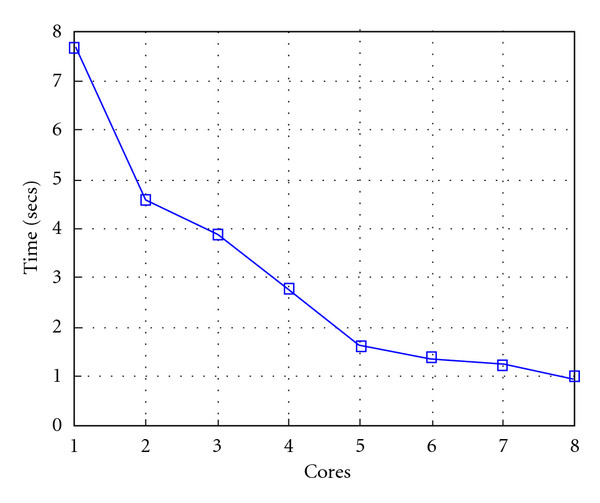
Test 1: Algorithm I: Total execution time versus the number of cores. Δ*τ*_*I*_ = 0.4.

**Figure 16:**
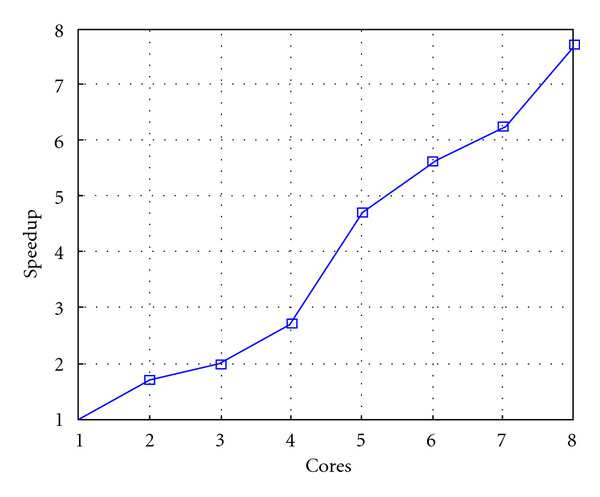
Test 1: Algorithm I: Speedup. Δ*τ*_*I*_ = 0.4.

**Figure 17:**
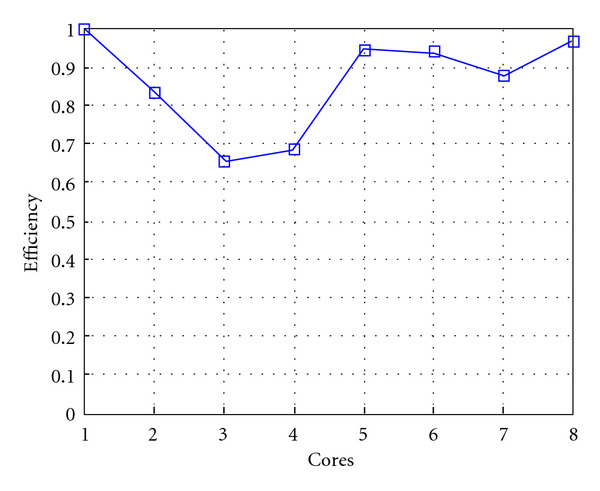
Test 1: Algorithm I: Efficiency. Δ*τ*_*I*_ = 0.4.

**Figure 18:**
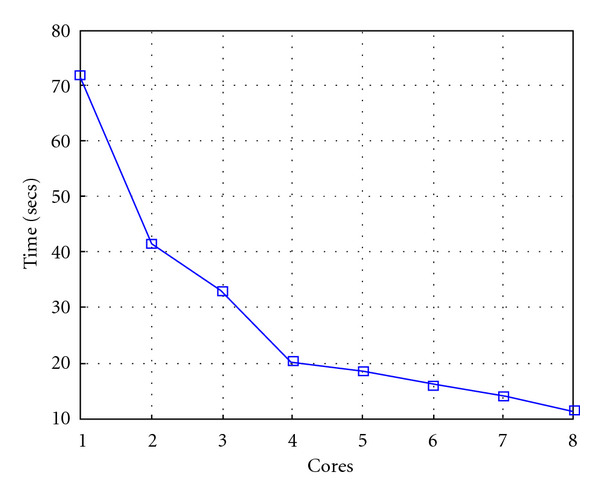
Test 1: Algorithm I: Total execution time versus the number of cores. Δ*τ*_*I*_ = 0.04.

**Figure 19:**
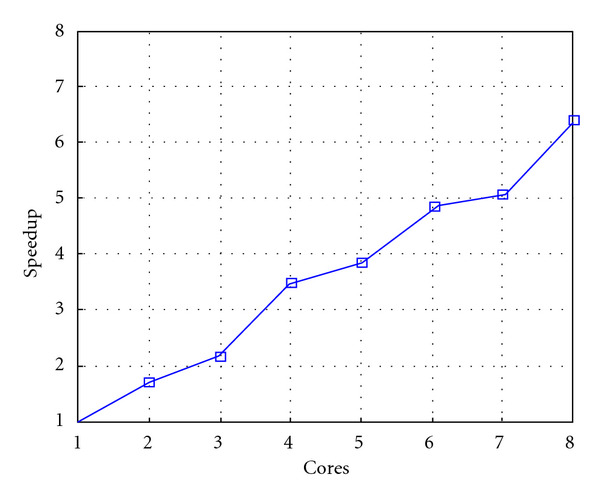
Test 1: Algorithm I: Speedup. Δ*τ*_*I*_ = 0.04.

**Figure 20:**
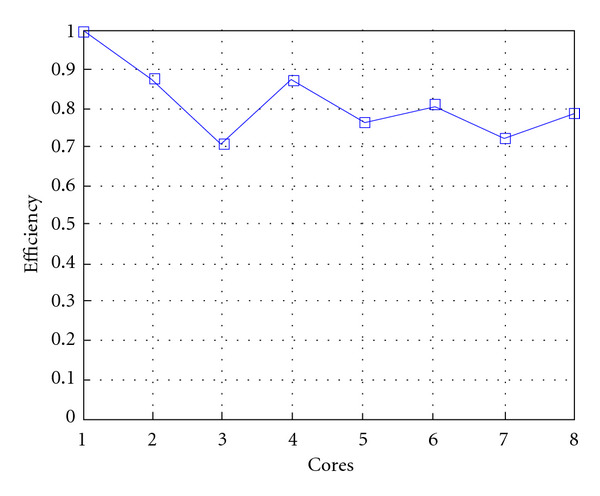
Test 1: Algorithm I: Efficiency. Δ*τ*_*I*_ = 0.04.

Finally, we report execution time, speedup, and efficiency of Algorithm I, at scale step Δ*τ* = 0.4 (i.e., Figures 15, 16 and 17) and Δ*τ* = 0.04 (i.e., Figures 18, 19, and 20), respectively. Note that parallel efficiency of both algorithms always is, at least, of 60% and, on average, of about 80%. In particular, parallel efficiency of Algorithm I is about 90%. Execution time of both algorithms reduces to about 2 seconds on eight cores in the first case (i.e., *E*_*d*_ = (10^−1^), and to about 10 seconds in the second case (*E*_*d*_ = *O*(10^−2^)). This means that, in a multicore computing environment, both algorithms provide the requested solution within a response time that can be considered quite acceptable in medical imaging applications and, in particular, that Algorithm I is competitive with Algorithm SI. 

**Figure 21:**
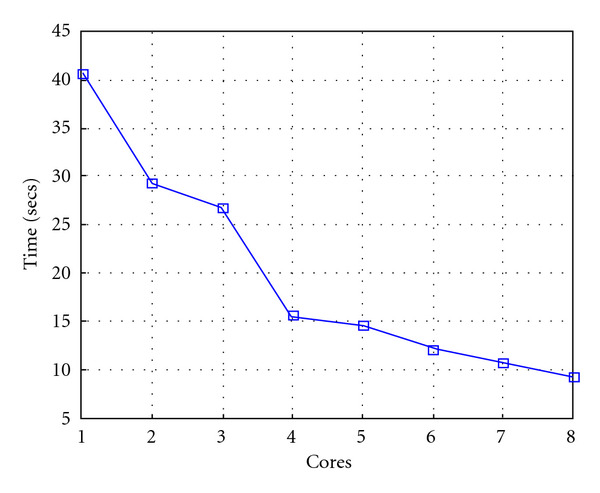
Test 2: Algorithm SI: Total execution time versus the number of cores. Δ*τ*_*I*_ = 0.16.

**Figure 22:**
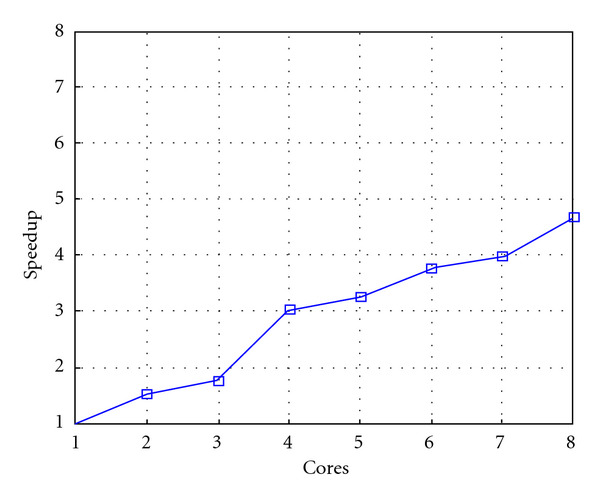
Test 2: Algorithm SI: Speedup. Δ*τ*_*I*_ = 0.16.

**Figure 23:**
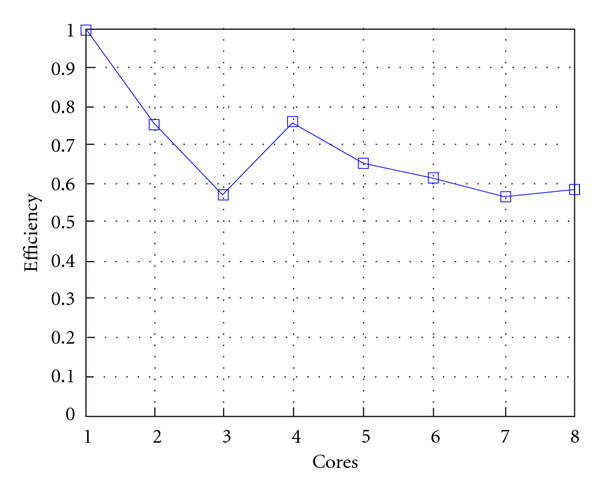
Test 2: Algorithm SI: Efficiency. Δ*τ*_*I*_ = 0.16.

**Figure 24:**
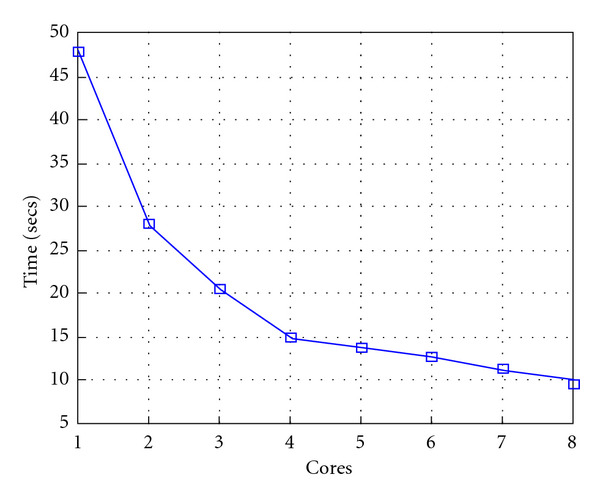
Test 2: Algorithm I: Total execution time versus the number of cores. Δ*τ*_*I*_ = 0.4.

**Figure 25:**
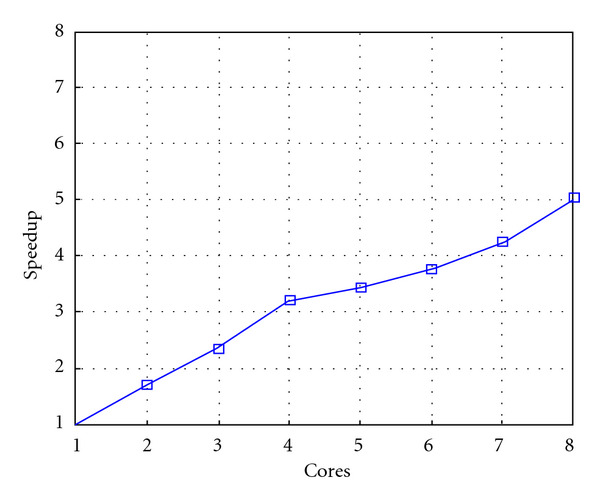
Test 2: Algorithm I. Speed up. Δ*τ*_*I*_ = 0.4.

**Figure 26:**
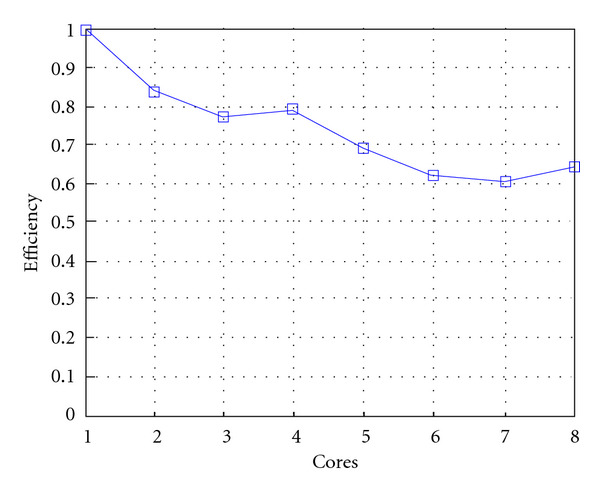
Test 2: Algorithm I. Efficiency. Δ*τ*_*I*_ = 0.4.

Figures 21, 22, 23, 24, 25, and 26 show time, speedup, and parallel efficiency of two algorithms in case of Test 2. We only consider the case of Δ*τ*_SI_ = 0.16 and Δ*τ*_*I*_ = 0.4.

Finally, we show results on scalability of parallel algorithms. Let *T*_*p*_(*N*) be the execution time of the parallel algorithm running on *p* cores for segmenting an image of size *N*^2^ × *N*^2^. We measure the scalability of these algorithms by measuring *T*_*p*_(*N*) as *N* varies, once *p* is fixed, and by measuring *T*_*p*_(*N*) as *N* and *p* grow. We note that, in case of Algorithm SI, the scaling factor is

(41) $$\frac{T_{p}\left(2N\right)}{T_{p}\left(N\right)}≃4.2,$$

while, for Algorithm I, we get as scaling factor

(42) $$\frac{T_{p}\left(2N\right)}{T_{p}\left(N\right)}≃2.3.$$

**Figure 27:**
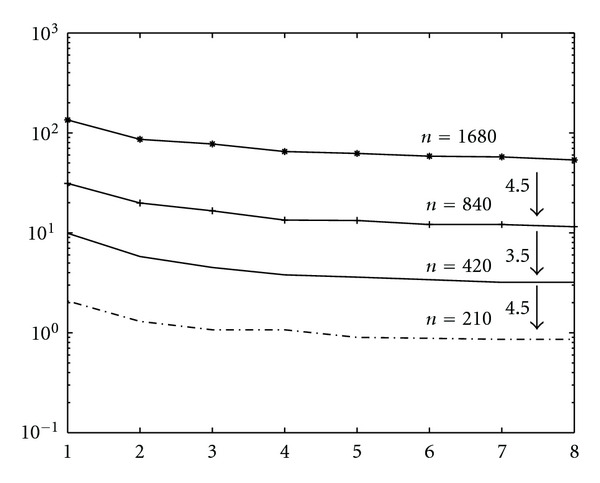
Scalability of parallel Algorithm SI, as *N* is fixed and *p* varies, and *N* = 210,420,840,1680. Each line refers to the execution time of the algorithm at a fixed value of *N*.

**Figure 28:**
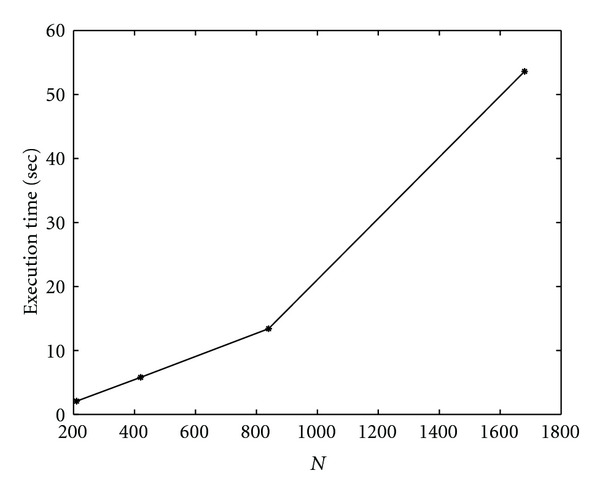
Scalability of parallel Algorithm SI, as *N* and *p* vary. Each point of the graph refers to the execution time of the parallel semi-implicit algorithm at (*p* = *k* · *p*_1_, *N* = *k* · *N*_1_), where *p*_1_ = 1, *N*_1_ = 210, and *k* = 1,2, 3,4.

**Figure 29:**
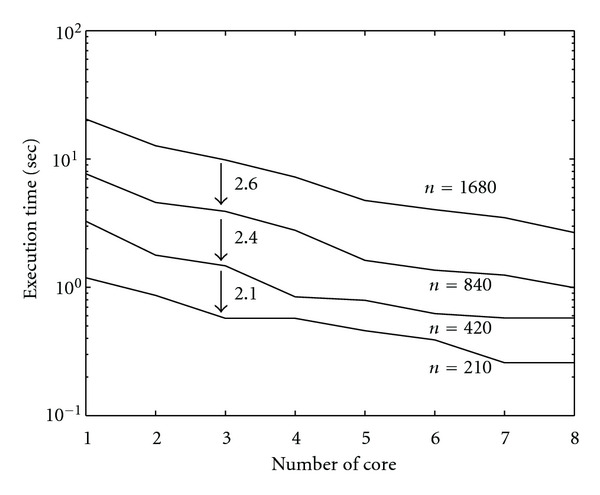
Scalability of parallel Algorithm I, as *N* is fixed and *p* varies, and *N* = 210,420,840,1680. Each line refers to the execution time of the algorithm at a fixed value of *N*. Δ*τ*_*I*_ = 0.4.

**Figure 30:**
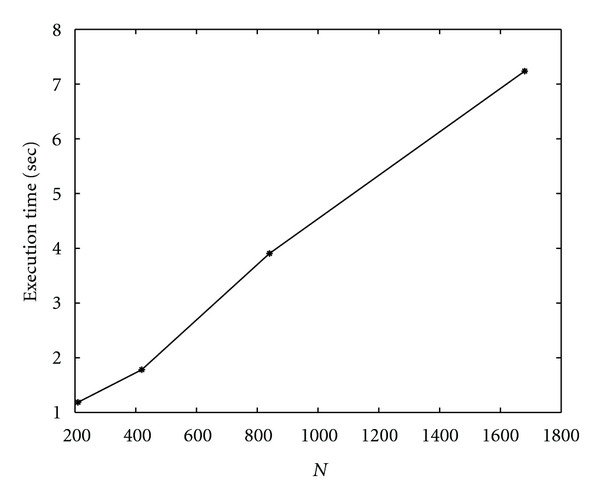
Scalability of parallel Algorithm I, as *N* and *p* vary. Each point of the graph refers to the execution time of the parallel implicit algorithm at (*p* = *k* · *p*_1_, *N* = *k* · *N*_1_), where *p*_1_ = 1, *N*_1_ = 210 and *k* = 1,2, 3,4. Δ*τ*_*I*_ = 0.4.

This means that Algorithm I scales better than Algorithm SI. In Figures 27 and 28 (for Algorithm SI) and Figure 29 and 30 (for Algorithm SI), we report *T*_*p*_(*N*) as *N* and *p* varies. In particular, each point of the graph refers to the execution time of the parallel algorithm at (*p* = *k* · *p*_1_, *N* = *k* · *N*_1_), where *p*_1_ = 1, *N*_1_ = 210, and *k* = 1,2, 3,4.

## 7. Conclusions

A straightforward comparison between the semi-implicit and the fully implicit discretization schemes of nonlinear PDE of parabolic/hyperbolic type states that fully implicit discretization usually leads to too expensive algorithms. In this paper, we provide a multicore implementation of two numerical algorithms arising from using these two discretization schemes: semi-implicit (Algorithm SI) and fully implicit (Algorithm I). Taking into account that we aim to solve such problems on parallel computer in a scalable way, in the first case, we use, as linear solver, Krylov iterative methods (GMRES) with algebraic multigrid preconditioners (AMG). Regarding the fully implicit scheme, we use the Jacobian-Free-Newton Krylov (JFNK) method as nonlinear solver. 

We compare these two algorithms using different metrics measuring both the accuracy and the efficiency. We note that if we require that the discretization error *E*_*d*_ is *E*_*d*_ = *O*(10^−1^), these two algorithms are quite numerically equivalent, both in terms of execution time and of the computed result; while, as discretization error decreases, Algorithm I appears to be more robust than Algorithm SI, in the sense that Algorithm I reaches the steady state with high accuracy (the Hausdorff distance is of 95%), while the computed results of Algorithm SI are less accurate, even though the execution time of Algorithm I sometimes slightly increases. These results suggest that if it needs to get accurate and reliable results, Algorithm I should be preferable.

 The parallel efficiency of both algorithms always is, at least, of 60% and, on average, of about 80%. In particular, parallel efficiency of Algorithm I is of about 90%. Execution time of both algorithms reduces to about 2 seconds on eight cores if *E*_*d*_ = (10^−1^) and to about 10 seconds if *E*_*d*_ = *O*(10^−2^). This means that, in a multicore computing environment, Algorithm I is competitive with Algorithm SI. 

In conclusion, our results suggest that if it is required high accuracy of the computed solution in a suitable turnaround time, using a multicore computing environment fully implicit scheme provides an accurate and reliable solution within a response time of few seconds, quite acceptable in medical imaging applications, such as computer-aided-diagnosis.

## Figures and Tables

**Algorithm 1:**
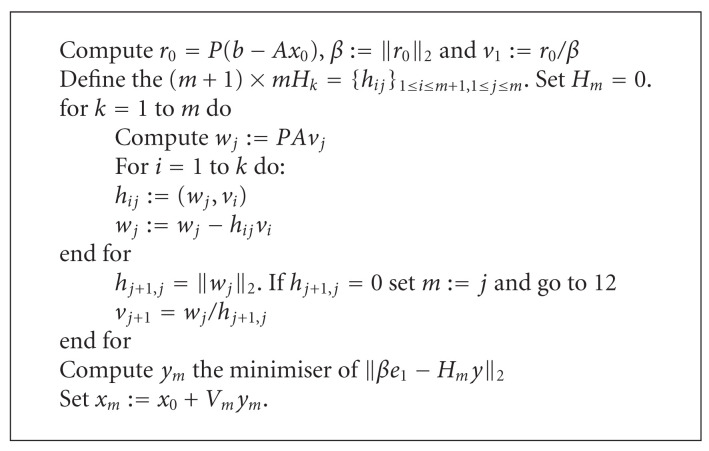
Preconditioned GMRES for solving a linear system *Ax* = *b*. Input: *A* (matrix coefficient), *b* (right hand side), *P* (preconditioner). Output: *x*_*m*_, approximate solution at the *m*th step. For all iteration, a matrix-vector product is required.

**Algorithm 2:**
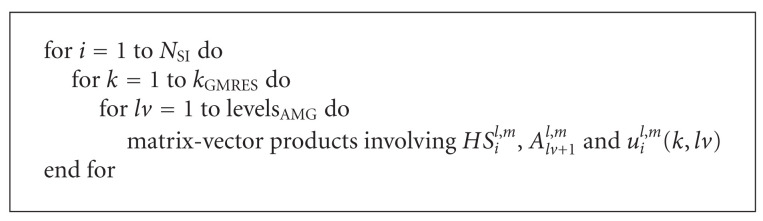
Sketch of Algorithm SI.

**Algorithm 3:**
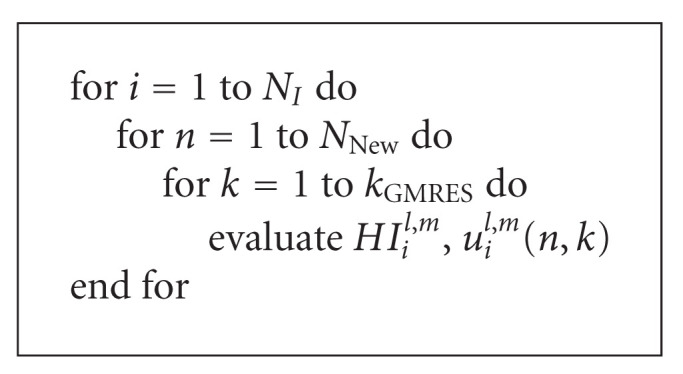
Sketch of Algorithm I.

**Table 1:** Comparisons between two algorithms: *E*_*d*_ = *O*(10^−1^).

Algorithm	Δ*τ*	Number of scale steps	*d*_*H*_	Execution time (secs)
SI	0.16	3	0.9492	9.262
I	0.4	1	0.9498	7.675

**Table 2:** Comparisons between two algorithms: *E*_*d*_ = *O*(10^−2^).

Algorithm	Δ*τ*	Number of scale steps	*d*_*H*_	Execution time (secs)
SI	0.016	25	0.6412	61.487
I	0.04	10	0.9498	71.67

**Table 3:** Comparisons between two algorithms: *E*_*d*_ = *O*(10^−3^).

Algorithm	Δ*τ*	Number of scale steps	*d*_*H*_	Execution time (secs)
SI	0.0016	250	0.5993	356.926
I	0.004	100	0.9492	356.335

**Table 4:** Convergence history of Algorithm SI. Δ*τ*_SI_ = 0.16. First column reports the scale step number, second column reports the number of GMRES iterations at each scale step, and last column reports the maximum number of AMG levels at each GMRES iteration.

*i*	*k*_gmres_^*i*^	*lv* ^*i*^
1	8	6
2	6	5
3	5	3

**Table 5:** Convergence history of Algorithm SI. Δ*τ*_SI_ = 0.016. On the first column, we denote the scale step number, and the symbol *p* − *q* is used to denote the steps ranging from the *p*-th until to the *q*-th. Second column reports the number of GMRES iterations at each scale step, third column reports the maximum number of AMG levels at each GMRES iteration.

*i*	*k*_gmres_^*i*^	*lv* ^*i*^
1	5	6
2-3	5	5
4	5	4
5–7	4	4
8-8	4	3
10–13	3	3
14–20	3	2
21-22	3	1
23–25	2	1

**Table 6:** Convergence history of Algorithm SI. Δ*τ*_*I*_ = 0.4. First column reports the scale step number, second column reports the number of Newton's steps at each scale step, and last column reports the number of GMRES iterations at each Newton's step.

*i*	*N*_New_^*i*^	*k*_GMRES_^*i*^
1	10	9, 9, 8, 8, 8, 7, 7, 7, 6, 6

**Table 7:** Convergence history of Algorithm SI. Δ*τ*_*I*_ = 0.04. First column reports the scale step number, second column reports the number of Newton's steps at each scale step, and last column reports the number of GMRES iterations at each Newton's step.

*i*	*N*_New_^*i*^	*k*_GMRES_^*i*^
1	10	9, 9, 8, 8, 7, 7, 7, 7, 6, 6
2	10	9, 8, 8, 8, 7, 7, 7, 6, 6, 6
3	9	9, 8, 8, 7, 7, 6, 6, 6, 6
4	9	8, 8, 8, 7, 7, 6, 6, 6, 5
5	9	8, 8, 7, 7, 7, 6, 6, 5, 5
6	7	8, 7, 7, 7, 6, 5, 5
7	7	7, 7, 7, 7, 6, 5, 5
8	6	7, 7, 6, 6, 6, 5
9	6	7, 7, 6, 6, 5, 5
10	5	7, 6, 6, 5, 4

**Table 8:** Test 2: Comparisons between two algorithms: *E*_*d*_ = *O*(10^−1^).

Algorithm	Δ*τ*	Number of scale steps	Execution time (secs)
SI	0.16	13	41.125
I	0.4	5	47.893

**Table 9:** Test 2: Comparisons between two algorithms: *E*_*d*_ = *O*(10^−2^).

Algorithm	Δ*τ*	Number of scale steps	Execution time (secs)
SI	0.016	125	417.56
I	0.04	50	455.33

**Table 10:** Test 2: Comparisons between two algorithms: *E*_*d*_ = *O*(10^−3^).

Algorithm	Δ*τ*	Number of scale steps	Execution time (secs)
SI	0.0016	1250	4828.5
I	0.004	500	4663.7
